# One-dimensional mean computed tomography value evaluation of ground-glass opacity on high-resolution images

**DOI:** 10.1007/s11748-012-0066-7

**Published:** 2012-06-01

**Authors:** Akihiko Kitami, Yoshito Kamio, Shoko Hayashi, Kosuke Suzuki, Shugo Uematsu, Ryozo Gen, Takashi Suzuki, Mitsutaka Kadokura

**Affiliations:** 1 Respiratory Disease Center, Showa University Northern Yokohama Hospital, 35-1 Chigasaki-Chuo, Tsuzuki-ku, Yokohama, 224-8503 Japan; 2 Division of Chest Surgery, Showa University School of Medicine, Tokyo, Japan

**Keywords:** Lung cancer, Bronchioloalveolar carcinoma, Ground-glass opacity, Mean computed tomography value, Follow-up

## Abstract

**Objective:**

Differentiation of atypical adenomatous hyperplasia (AAH), bronchioloalveolar carcinoma (BAC), and invasive carcinoma on computed tomography (CT) is useful for determining “follow-up or resection” strategies for lesions displaying ground-glass opacity (GGO). The purpose of this study is to evaluate one-dimensional quantitative CT values of GGO on high-resolution CT (HRCT) images using computer-aided diagnosis.

**Methods:**

Between April 2001 and March 2010, a total of 44 nodules in 42 patients with pure or mixed GGOs ≤2 cm were retrospectively evaluated. Maximum diameter and one-dimensional mean CT (m-CT) value of the diameter were measured using a computer graphics support system (HOPE/DrABLE-EX, Fujitsu, Tokyo, Japan) that displays a CT density profile across the tumor.

**Results:**

m-CT values were −682 ± 64 HU (range) for AAH lesions, −544 ± 179 (range) *for Type* A lesions, −496 ± 147 (range) for Type B lesions, and −371 ± 142 (range) for invasive lesions. AAH lesions had a significantly lower m-CT value than Type B lesions. AAH, Type A, and Type B lesions had significantly lower m-CT values than invasive lesions (*p* < 0.05). All seven GGO lesions with a maximum diameter ≤1 cm and m-CT value ≤−600 HU were pre-invasive lesions, while 16 of 22 (73 %) cases with maximum diameter >1 cm and m-CT value >−600 HU were invasive lesions.

**Conclusion:**

Observation may be indicated for GGO lesions with a maximum diameter ≤1 cm and m-CT value ≤−600 HU.

## Introduction

Recent advances in computed tomography (CT) scanning technology have enabled the detection of many lung cancers, particularly adenocarcinoma, at an earlier and potentially more curable stage than was previously possible [1]. However, the true clinical significance of small tumors found through screening is unknown, as no differences in mortality between screened and unscreened individuals have been observed [2, 3]. Thus, treatment strategies for small tumors displaying ground-glass opacity (GGO) remain controversial. The purpose of this study is to evaluate the one-dimensional quantitative CT value of GGO on high-resolution CT (HRCT) images using computer-aided diagnosis to retrospectively select “follow-up or resection” strategies for GGO.

## Patients and methods

Between April 2001 and March 2010, a total of 54 nodules with GGO in 52 patients were resected at our hospital. During this period, we recommended resection of GGO lesions that did not decrease in size during 3 months of follow-up. Pathologic specimens demonstrated adenocarcinoma in 47 nodules, atypical adenomatous hyperplasia (AAH) in 4 nodules, and lymphoproliferative disorder in 1 nodule. Of these, 44 nodules in 42 patients with pure or mixed GGOs ≤2 cm in size were evaluated in this retrospective analysis.

Pure GGO was defined as a shadow completely occupied by a hazy increased attenuation of the lung, with reservation of the bronchial and vascular margins in the lesion with no solid regions on HRCT. Mixed GGO was defined as a heterogeneous attenuation with a solid portion obscuring the underlying vascular markings.

Un-enhanced CT scans were performed from lung apices to bases during breath holding at mid-inspiration using a CT scanner (Aquilion 64, Toshiba, Tokyo, Japan). The helical scanning protocol was as follows: 120 kVp; 200 mA; 0.5-s scanning time; window level, −500 Hounsfield units (HU); window width, 1.600 HU; and a 512 × 512 matrix corresponding to a pixel size of approximately 0.6 mm. HRCT images were reconstructed at 0.5 mm intervals with a high-spiral-frequency algorithm (bone algorithm).

**Fig. 1:**
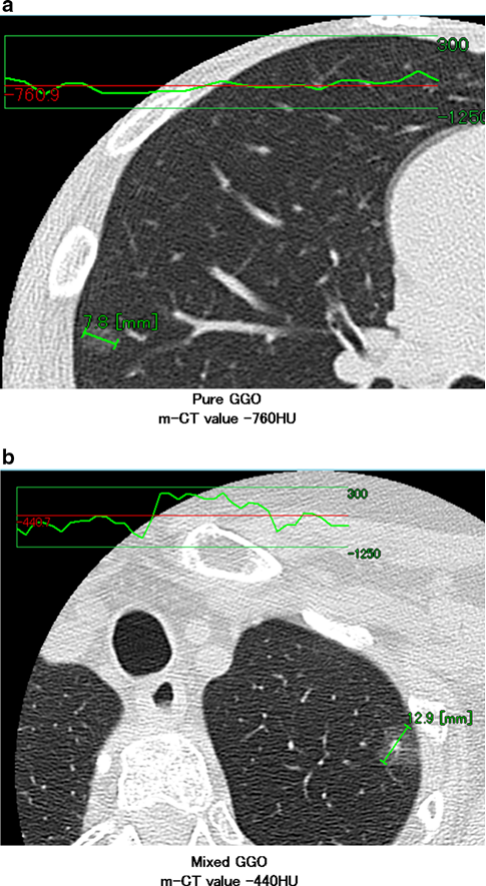
The CT image with a profile of the density across the tumor. The profile of pure GGO revealed an almost flat curve, and the m-CT value was calculated to be −760 HU (**a**). The profile of mixed GGO revealed that the CT value of the solid portion was higher than the GGO portion, and the m-CT value was calculated to be −440 HU (**b**)

Maximum diameter and one-dimensional mean CT (m-CT) value of this diameter were measured using a computer graphics support system (HOPE/DrABLE-EX. Fujitsu, Tokyo, Japan) that displays a CT density profile across the tumor (Fig. 1).

Histologic findings of adenocarcinomas were classified according to the criteria of the World Health Organization [4] and the criteria of Noguchi et al. [5]. The classification system for the replacement of growth patterns developed by Noguchi et al. is as follows: Type A (localized bronchioloalveolar carcinoma LBAC), Type B (LBAC with foci of collapse of alveolar structure), Type C (LBAC with foci of active fibroblastic proliferation), Type D (poorly differentiated adenocarcinoma), Type E (tubular adenocarcinoma), Type F (papillary adenocarcinoma with compressive and destructive growth). AAH was defined as a proliferation of minimally atypical cuboidal type II pneumocytes with gaps between the cells. We classified AAH and Type A and B as to pre-invasive lesions, and type C, D, E, and F as invasive lesions.

All data were expressed as mean ± standard deviation (SD). The differences in mean and SD values were analyzed between groups using a two-tailed Student’s *t* test. Between-group differences with *p* values <0.05 were regarded as significant.

## Results

Patients ranged in age from 39 to 83 years (median 62 years). Thirty patients were female and twelve were male. Radiologic findings showed pure GGO in 22 nodules and mixed GGO in 22 nodules. The maximum tumor dimension was ≤10 mm in 19 nodules, 11–15 mm in 11 nodules, and 16–20 mm in 14 nodules. The m-CT values were −559 ± 133 HU for pure GGO and −355 ± 144 HU for mixed GGO. The mean interval from first detection of focal GGO to pulmonary resection was 11.9 months (range 3–48 months). Surgical procedures included lobectomy in 19 patients, segmentectomy in 6 patients, and partial resection in 19 patients. The pathologic specimens demonstrated adenocarcinoma in 22 nodules, bronchioloalveolar carcinoma (BAC) in 18 nodules, and AAH in 4 nodules. Pure GGOs consisted of 16 pre-invasive lesions (Type A, Type B, and AAH) and 6 invasive lesions (Types C, D, E, and F), while mixed GGOs consisted of 6 pre-invasive lesions and 16 invasive lesions. None of the 27 patients who underwent lymph node dissection or sampling had lymph node involvement. All patients, except for 1 died from other disease, remain alive with no evidence of tumor recurrence to date, with a median follow-up of 41 months.

**Fig. 2:**
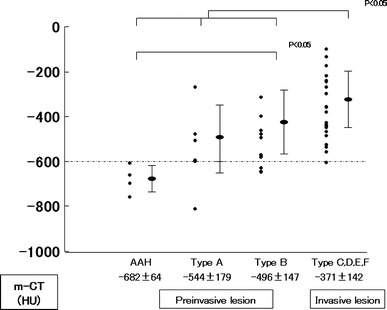
The m-CT values were −682 ± 64 HU (range) for AAH lesions, −544 ± 179 (range) for Type A lesions, −496 ± 147 (range) for Type B lesions, and −371 ± 142 (range) for invasive lesions. AAH lesions had a significantly lower m-CT value than Type B lesions. AAH, Type A, and Type B lesions had a significantly lower m-CT values than invasive lesions (*p* < 0.05). No significant differences in m-CT values were observed between AAH and Type A lesions or between Type A and Type B lesions. The m-CT value of AAH lesions were all ≤−600 HU, while most of the m-CT values of invasive lesions were >−600 HU

Figure 2 illustrates the distributions of m-CT value of maximum diameters for pre-invasive lesions (AAH, Type A, and Type B) and invasive lesions (Types C, D, E, and F). The m-CT value was −682 ± 64 HU (range) for AAH lesions, −544 ± 179 (range) for Type A lesions, −496 ± 147 (range) for Type B lesions, and −371 ± 142 (range) for invasive lesions. AAH had a significantly lower m-CT value than Type B. AAH, Type A, and Type B lesions had significantly lower m-CT values than invasive lesions (*p* < 0.05). No significant differences in m-CT values were observed between AAH and Type A lesions or between Type A and Type B lesions. The m-CT values of AAH lesions were all ≤−600 HU; in contrast, most of the m-CT values for invasive lesions were >−600 HU. Based on these findings, a CT value of −600 HU appears to represent a cutoff value between AAH and invasive lesions.

**Fig. 3:**
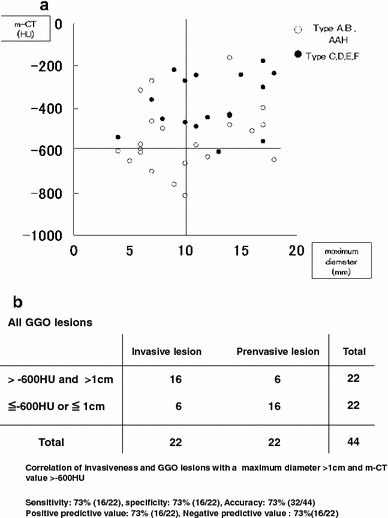
**a**, **b** Relationship between m-CT value and maximum diameter of all GGO lesions. All seven GGO lesions with a maximum diameter ≤1 cm in the maximum diameter and m-CT value ≤−600 HU were pre-invasive lesions, while 16 of 22 (73 %) with a maximum diameter >1 cm and m-CT value >−600 HU in m-CT were invasive lesions

Figure 3 illustrates the relationship between m-CT value and maximum diameter of all GGO lesions. All seven GGO lesions with a maximum diameter ≤1 cm and m-CT value ≤−600 HU were pre-invasive lesions, while 16 of 22 (73 %) cases with a maximum diameter >1 cm and m-CT value >−600 HU were invasive lesions (a).

m-CT value and maximum diameter of GGO lesions are significant predictive factors of invasiveness of the tumor. GGO lesions with a maximum diameter >1 cm and m-CT value >−600 HU are highly diagnosed as invasive lesions. The diagnostic accuracy is 73 % (32/44) (sensitivity 73 %, specificity 73 %, positive predictive value 73 %, negative predictive value 73 %) (b).

**Fig. 4:**
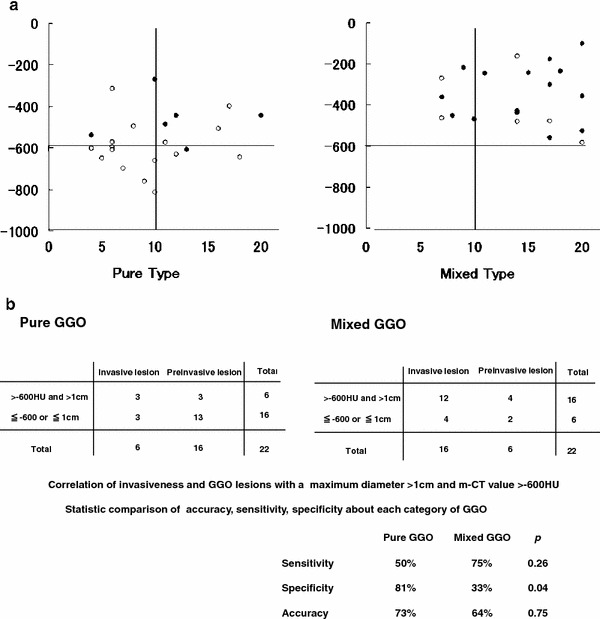
**a**, **b** Relationship between m-CT value and maximum diameter of pure and mixed type GGO lesions. GGO lesions ≤1 cm in maximum diameter and ≤−600 HU in m-CT value are all pure type. Although, GGO lesions of both categories with a maximum diameter >1 cm and m-CT value >−600 HU are highly diagnosed as invasive lesions, specificity of pure GGO were significantly better than that of mixed GGO

Figure 4 illustrates the relationship between m-CT value and maximum diameter of pure GGO and mixed GGO. All GGO lesions with a maximal diameter ≤1 cm and ≤−600 HU are pure type (a). Although, GGO lesions of both categories with a maximum diameter >1 cm and m-CT value >−600 HU are highly diagnosed as invasive lesions, specificity of pure GGO were significantly better than that of mixed GGO (b).

## Discussion

GGO is defined as a shadow completely occupied by a hazy increased attenuation of the lung, with reservation of the bronchial and vascular margins in the lesion on HRCT. Although GGO is a nonspecific finding that may be caused by various disorders, including inflammatory disease, fibrosis, and neoplastic disease [6], the pathologic diagnoses of GGO in this study, adenocarcinoma in 47 patients (90 %) and AAH in 4 patients (8 %), were shown to be neoplastic lesions, and all patients with adenocarcinoma had T1N0M0 disease. These results suggest that persistent focal GGO is a significant sign of early-stage adenocarcinoma.

Many radiologic studies of small lung adenocarcinomas have demonstrated a strong correlation between CT findings and pathologic features. Several authors have classified small lung lesions into nonsolid (pure) GGO type, partly solid (mixed) GGO type, and solid type, and have suggested that pure type and mixed type with small solid components are almost always non-invasive carcinomas [7–10]. However, it is sometimes difficult to differentiate between pure and mixed GGO, and between high-density GGO and solid tumor. Suzuki et al. [11] classified homogeneous (so-called “pure”) GGO into pure GGO and semiconsolidation to evaluate the differences in density within the tumor, and stated that pathologically, semiconsolidation tends to be adenocarcinoma with invasive foci. Although the differentiation between pure GGO and semiconsolidation is unclear in their report, density within homogeneous GGO appears to be an important factor for predicting tumor invasiveness.

Some authors have used quantitative densitometric methodologies to evaluate GGO lesions [12–15]. Ikeda et al. reported that the 75th percentile CT value analyzed by three-dimensional computerized quantification of GGO lesions was the optimal CT value for differentiating between AAH, BAC and adenocarcinoma. These investigators noted that CT cutoff value of −584 HU was optimal for differentiation between AAH and BAC, and −472 HU was optimal for differentiation between BAC and adenocarcinoma [13]. Nomori et al. used histograms of CT pixel numbers for AAH and nonmucinous adenocarcinoma to quantify peaks and mean numbers of CT pixels, and found the mean value of mean CT values to be −697 ± 56 HU for AAH lesions and −541 ± 73 for BAC lesions. These investigators noted that the peak CT value on the histogram is the most frequent value observed in the tumor, and that the effect of vessels and bronchi within the tumor can be ignored [14]. Although the one-dimensional quantitative m-CT value can be slightly affected by the densities of vessels or bronchi within the tumor, this calculation method is straightforward, and can similarly estimate pure GGO and mixed GGO. The present study demonstrates that an m-CT value of −600 HU may represent a cutoff value demarcating AAH versus invasive lesions. We believe that this finding could significantly impact the selection of therapeutic strategies for GGO lesions.

Kushihashi et al. [16] reported CT findings of AAH as bronchioloalveolar adenoma in 1994. Although AAH is usually discovered incidentally during microscopic examination of surgically resected lung specimens, a recent increase in the detection of AAH as pure GGO has occurred. Pathologically, AAH was classified as a premalignant lesion in the 1999 World Health Organization classification. Thus, while the clinical and pathological features of AAH have become clearer, therapeutic strategy for AAH lesions have not. AAH is considered to be a precursor of BAC or adenocarcinoma [17–19]; however, no reports of progression of AAH to BAC or adenocarcinoma have been published. We believe that at time, pure GGO lesions that are suspected to be AAH based on m-CT values are now possible to follow-up.

Whether GGO lesions that are suspected to be BAC should be resected or followed up also remains controversial. Recently, several researchers reported that for noninvasive lesions identified on HRCT, careful observation without surgical intervention represents a valid treatment options [20–22]. According to the interim guidelines suggested by Godoy and Nadich [23], isolated lesions with pure GGO that are ≤5 mm do not necessarily require CT follow-up, since they almost always represent foci of AAH, but for lesions between 5 and 10 mm, follow-up is requisite pending better definition of their true nature; lesions >1 cm should be assumed to be BAC or invasive adenocarcinoma, but surgery should be considered particularly if the nodule is growing or if an increase in attenuation or development of a solid component is observed.

Diligent long-term follow-up is the natural history of pure GGO, and should be conducted to determine whether surgical intervention is acceptable or unnecessary [24]. However, we believe that early detection and early therapy of some primary lung cancers with GGO is important for improving individual prognoses for the following reasons. First, pure GGO lesions are not all histologically pre-invasive lesions, as some have an invasive adenocarcinoma component, such as Noguchi’s Type C. Second, some researchers have reported the concept of a multi-step progression from AAH through localized BAC (type A and B) to advanced adenocarcinoma with a replacement growth pattern (Type C) [17–19]. Third, patients with Noguchi’s type C lesions have a worse 5-year survival rate (75 %) than patients with type A or B lesions (100 %).

GGO lesions with a maximum diameter >1 cm and m-CT value >−600 HU are absolute indication of operation, because those lesions were highly diagnosed as invasive lesions. GGO lesions with a maximum diameter ≤1 cm and m-CT ≤−600 HU should be observed, because all seven lesions were preinvasive lesions. Although, GGO lesions with a maximum diameter >1 cm or m-CT value >−600 HU are possible to be observed, we think these lesions should be resected because it is more important to avoid observation for invasive lesions than resection for preinvasive lesions.

It is noteworthy that pure GGO are not all preinvasive lesions, and specificity of pure GGO correlated between invasiveness and maximum diameter and m-CT value is significantly higher than that of mixed GGO. Evaluation of m-CT value is useful for determining “follow up or resection” for GGO, especially pure type.

The usefulness of m-CT value for benign GGO including inflammatory disease, fibrosis, and so on is unclear, because 98 % of removed GGO in this study is malignant neoplasm including AAH.

We think persistent GGO is almost all neoplasms, so differentiating benign and malignant is more important solid lesions than GGO lesions.

## Conclusion

Evaluation of m-CT value is useful for determining “follow up or resection” for GGO, especially pure type. Although the number of cases is small, all lesions with a maximum diameter ≤1 cm and m-CT ≤−600 HU are pre-invasive lesions. Therefore, observation may be indicated for this type of GGO lesion.

## References

[1] Henschke CI, McCauley DI, Yankelevitz D, Naidich DP, McGuinness G, Miettinen OS (1999). Early lung cancer action project: overall design and findings from baseline screening. Lancet.

[2] Parkin DM, Moss SM (2000). Lung cancer screening. Improved survival but no reduction in deaths—the role of “overdiagnosis”. Cancer.

[3] Patz EF, Goodman PC, Bepler G (2000). Screening for lung cancer. N Engl J Med.

[4] Travis WD, Colby TV, Corrin B, Shimosato Y, Brambilla E, Sobin LH (1999). Histological typing of lung and pleural tumors. World Health Organization international histological classification of tumors.

[5] Noguchi M, Morikawa A, Kawasaki M, Matsuno Y, Yamada T, Hirohashi S (1995). Small adenocarcinoma of the lung. Histologic characteristics and prognosis. Cancer.

[6] Collins J, Stern EJ (1997). Ground-glass opacity at CT: the ABCs. Am J Roentgenol.

[7] Kodama K, Higashiyama M, Yokouchi H, Takami K, Kuriyama K, Mano M (2001). Prognostic value of ground-glass opacity found in small lung adenocarcinoma on high-resolution CT scanning. Lung Cancer.

[8] Takashima S, Maruyama Y, Hasegawa M, Yamanda T, Honda T, Kadoya M (2002). Prognostic significance of high-resolution CT findings in small peripheral adenocarcinoma of the lung: a retrospective study on 64 patients. Lung Cancer.

[9] Nakata M, Saeki H, Takata I, Segawa Y, Mogami H, Mandai K (2002). Focal ground-glass opacity detected by low-dose helical CT. Chest.

[10] Matsuguma H, Nakahara R, Anraku M, Kondo T, Tsuura Y, Kamiyama Y (2004). Objective definition and measurement method of ground-glass opacity for planning limited resection in patients with clinical stage IA adenocarcinoma of the lung. Eur J Cardiothorac Surg.

[11] Suzuki K, Kusumoto M, Watanabe S, Tsuchiya R, Asamura H (2006). Radiologic classification of small adenocarcinoma of the lung: radiologic–pathologic correlation and its prognostic impact. Ann Thorac Surg.

[12] Yang ZG, Sone S, Takashima S, Li F, Honda T, Maruyama Y (2001). High-resolution CT analysis of small peripheral lung adenocarcinoma revealed on screening helical CT. AJR.

[13] Ikeda K, Awai K, Mori T, Kawanaka K, Yamashita Y, Nomori H (2007). Differential diagnosis of ground-glass opacity nodules. CT number analysis by three dimensional computerized quantification. Chest.

[14] Nomori H, Ohtsuka T, Naruke T, Suemasu K (2003). Differentiating between atypical adenomatous hyperplasia and bronchioloalveolar carcinoma using the computed tomography number histogram. Ann Thorac Surg.

[15] Yanagawa M, Kuriyama K, Kunitomi Y, Tomiyama N, Honda O, Sumikawa H (2009). One-dimensional quantitative evaluation of peripheral lung adenocarcinoma with or without ground-glass opacity on thin-section CT images using profile curve. Br J Radiol.

[16] Kushihashi T, Munechika H, Ri K, Kubota H, Ukisu R, Satoh S (1994). Bronchioloalveolar adenoma of the lung: CT-pathologic correlation. Radiology.

[17] Noguchi M, Shimosato Y (1995). The development and progression of adenocarcinoma of the lung. Cancer Treat Res.

[18] Kitamura H, Kameda Y, Nakamura M, Nakatani Y, Inayama Y, Iida M (1995). Proliferative potential and p53 overexpression in precursor and early stage lesions of bronchioloalveolar lung carcinoma. Am J Pathol.

[19] Takashima S, Maruyama Y, Hasegawa M, Yamanda T, Honda T, Kadoya M (2003). CT findings and progression of small peripheral lung neoplasms having a replacement growth pattern. AJR.

[20] Suzuki K, Asamura H, Kusumoto M, Kondo H, Tsuchiya R (2002). “Early” peripheral lung cancer: prognostic significance of ground-glass opacity on thin-section computed tomographic scan. Ann Thorac Surg.

[21] Kakinuma R, Ohmatsu H, Kaneko M, Kusumoto M, Yoshida J, Nagai K (2004). Progression of focal pure ground-glass opacity detected by low-dose helical computed tomography screening for lung cancer. J Comput Assist Tomogr.

[22] Asamura H, Suzuki K, Watanabe S, Matsuno Y, Maeshima A, Tsuchiya R (2003). A clinicopathological study of resected subcentimeter lung cancers: a favorable prognosis for ground-glass opacity lesions. Ann Thorac Surg.

[23] Godoy MCB, Nadich DP (2009). Subsolid pulmonary nodules and the spectrum of peripheral adenocarcinoma of the lung: Recommended interim guidelines for assessment and management. Radiology.

[24] Kodama K, Higashiyama M, Yokouchi H, Takami K, Kuriyama K, Kusunoki Y (2002). Natural history of pure ground-glass opacity after long-term follow-up of more than 2 years. Ann Thorac Surg.

[25] Aoki T, Nakata H, Watanabe H, Nakamura K, Kasai T, Hashimoto H (2000). Evolution of peripheral lung adenocarcinomas: CT findings correlated with histology and tumor doubling time. AJR.

